# Neurological and Psychiatric Disorders in Patients with Rheumatic Heart Disease: Unveiling what is Beyond Cardiac Manifestations

**DOI:** 10.5334/gh.1149

**Published:** 2022-08-29

**Authors:** Luiz Paulo Bastos Vasconcelos, Marcelle Cristina da Silva Bastos Vasconcelos, Francisco Biagio Murta E. Di Flora, Flávio Augusto Paes de Oliveira, Pedro Drummond Lima, Lucas Campos Barbosa E. Silva, Breno Camargos Mucelli Spolaor, José Luiz Padilha da Silva, William Antônio de Magalhães Esteves, Maria Carmo P. Nunes, Antônio Lúcio Teixeira

**Affiliations:** 1 Post Graduate Program in Infectious Diseases and Tropical Medicine, School of Medicine, Universidade Federal de Minas Gerais, Belo Horizonte, MG, Brazil; 2 Department of Statistics, Universidade Federal do Paraná, Curitiba, PR, Brazil; 3 Faculdade Santa Casa BH, Belo Horizonte, MG, Brazil; 4 The Neuropsychiatry Program, UT Health Science Center, Houston, Texas, US

**Keywords:** Rheumatic heart disease, basal ganglia motor dysfunction, obsessive-compulsive symptoms, mood disorders, migraine

## Abstract

**Background:**

Rheumatic heart disease (RHD) is the most serious manifestation of rheumatic fever, which may also affect the brain. The current study assessed the prevalence of neuropsychiatric manifestations in patients with RHD, including clinical features associated with basal ganglia motor dysfunction (BGMD).

**Methods:**

We conducted neurologic and psychiatric assessments in consecutive patients with RHD referred to a tertiary center for heart valve diseases. Echocardiography was performed to assess the pattern of valvular involvement and RHD severity. Validated questionnaires for the evaluation of cognition, depression, anxiety, and obsessive-compulsive symptoms (OCS) were applied. BGMD was clinically defined by the presence of hyperkinetic movement disorders.

**Results:**

Fifty patients with age of 43.2 ± 10.8 years, 84% female, were included. Mitral valve was affected in 47 patients (94%), and 21 of them (42%) also had aortic valve involvement. Chorea (22%), chronic tics (18%), OCS (48%), major depression (34%), generalized anxiety disorder (54%), cognitive complaints (66%), migraine (52%) and seizures (18%) were frequently reported. The factors associated with BGMD were age (p = 0.018), major depression (p = 0.013), and Yale-Brown Obsessive Compulsive (Y-BOCS) score (p = 0.011). The severity of heart disease was not associated with BGMD.

**Conclusions:**

Neuropsychiatric manifestations are frequent in RHD patients, which may persist up to three decades after acute rheumatic fever. Age, major depression and severity of OCS were independently associated with BGMD. These manifestations deserve a close attention of clinicians and researchers dealing with adult patients with RHD.

## 1. Introduction

Rheumatic heart disease (RHD) is an inflammatory disease characterized by autoimmune reaction to the heart valves associated with single or recurrent episodes of rheumatic fever (RF) [1]. RHD is the most serious manifestation of RF, at times resulting in heart failure, atrial fibrillation and stroke [2]. Although there is a global decline in RHD-related mortality, RHD remains a major cause of acquired cardiovascular diseases among children and young adults in low-income countries [3].

Sydenham’s chorea (SC) is the main neurological manifestation of RF and usually occurs in association with cardiac and articular symptoms. Up to 70% of patients with SC may present with concomitant carditis. SC is an autoimmune condition supposedly resulting from basal ganglia dysfunction triggered by cross-reactive antibodies produced during group A beta-hemolytic streptococcus (GABHS) pharyngitis [4]. The symptoms usually begin four to eight weeks after GABHS infection, and include chorea and other motor and behavioral symptoms [5]. Obsessive-compulsive symptoms (OCS), anxiety and depressive disorders are among the most common behavioral manifestations [6]. Migraine, and cognitive impairment have also been reported in SC, notably in children and young adults [5,6,7,8,9,10,11].

Although RHD involves mainly the heart valves, patients with RHD might also present persistent neurological and behavioral symptoms [12,13,14]. Actually, RHD patients may manifest these symptoms even without chorea, in other words, without a definitive diagnosis of SC [12,13,14,15]. However, data regarding the prevalence and impact of neurological and psychiatric disorders on social functioning and quality of life of adults with RHD are lacking [13].

We aimed to investigate the prevalence of chorea and other neurological and psychiatric disorders in patients with RHD, and whether they are associated with the severity of the related heart disease. We also aimed to evaluate which features are associated with clinical signs of basal ganglia motor dysfunction (BGMD) in patients with RHD.

## 2. Methods

### 2.1 Study design and population

We performed a cross-sectional assessment of all patients between 18 and 65 years-old with RHD referred to our tertiary center for heart valve diseases, from July 2018 to May 2019. Our center currently follows around 590 patients with RHD. Since many of the established patients are already under neuropsychiatric care, to avoid selection bias we included only newly referred patients who met the inclusion and exclusion criteria.

The exclusion criteria included patients with known cognitive impairment, and those who had a stroke. The latter were excluded as the related brain dysfunction could independently affect neurological and behavioral status [16].

The diagnosis of RHD was based on standard clinical and echocardiographic criteria [17]. Medical history with assessment of NYHA functional class, physical examination and routine laboratory tests were obtained in all patients. All patients had a transthoracic echocardiogram performed according to American Society of Echocardiography recommendations to assess morphological and functional aspects of the valves, and to obtain measurements and hemodynamic calculations [18]. The severity of valvular dysfunction was categorized into mild, moderate and severe [19]. Twelve-lead electrocardiogram was performed to assess cardiac rhythm and detect chronic atrial fibrillation (AF).

Neurological and behavioral assessment consisted in systematic evaluation of current and past medical and psychiatric history alongside neurologic exam and application of validated questionnaires for the evaluation of cognition, depression and anxiety and OCS [20,21,22,23,24]. The presence of chorea and tics was investigated through neurologic examination. The search for past history of SC was also undertaken in medical charts. Brain computerized tomography (CT) or magnetic resonance imaging (MRI) scans were performed in patients who had focal and/or sudden neurological deficits to exclude the occurrence of stroke.

The measurement of health-related quality of life was done using the Brazilian version of 12-Item Short-Form Health Survey Version 2 (SF-12v2) [25]. The SF-12v2 encompasses eight subscales grouped into two larger dimensions: the Physical Component Summary (PCS) and Mental Component Summary (MCS). For both PCS and MCS, the score ranges from 0 to 100 points, where higher scores mean better quality of life.

The study protocol was approved by the institutional ethics committee, and all enrolled patients gave written informed consent.

### 2.2. Definitions of neurological and psychiatric parameters

The definition of chorea and tics followed standard clinical criteria [5,26]. Basal ganglia motor dysfunction (BGMD) was clinically defined by the presence hyperkinetic movement disorders associated with RF, suggesting the involvement of frontostriatal motor circuit, in other words patients with well-documented history of SC during childhood or adolescence, and patients with current chorea and/or tics on examination [5,27].

Seizures were defined according to the International League Against Epilepsy (ILAE) 2017 seizure classification subtypes [28]. The diagnosis of migraines was done according to the criteria of the International Classification of Headache Disorders, 3^rd^ edition (ICHD-3) [29]. Cognitive complaints consisted of subjective complaints about difficulties in attention, memory and execution/planning of daily tasks reported by the patients [30].

The diagnosis of major depressive disorder and generalized anxiety disorder was assigned for patients currently meeting the DSM-5 criteria [26]. Obsessive-compulsive symptoms (OCS) referred to recurrent or persistent thoughts experienced as intrusive and/or repetitive behaviors or mental acts done in response to obsessions. The Hospital Anxiety and Depression Scale (HADS) was used to quantify anxiety and depressive symptoms, and the Yale-Brown Obsessive-Compulsive Scale (Y-BOCS) was applied to measure the severity of OCS [22,23].

The overall cognitive function was assessed using the Brazilian version of the Mini-Mental State Examination (MMSE), while frontal-executive functions were assessed using the Brazilian version of the Frontal Assessment Battery (FAB) [20,21]. We defined patients with low MMSE score as those performing lower than the proposed MMSE cutting-off scores adjusted for years of schooling [20].

### 2.3. Statistical analysis

Continuous data were expressed as mean ± s.d. or as median and interquartile range, depending on normality distribution. Categorical data were summarized as numbers and percentages. The variables of RHD patients with or without BGMD were compared using Fisher exact test, unpaired Student’s t test or Mann-Whitney U test, as appropriate. Logistic regression analysis was performed to determine which variables were independently associated with BGMD. Demographic, neurological and psychiatric variables that were found to be significantly associated with BGMD in univariate analysis were included in the multivariable logistic regression analysis. The potential predictive variables of BGMD included in multivariate analysis were age, migraine, generalized anxiety disorder, major depressive disorder and Y-BOCS. A value of p < 0.05 was considered significant.

## 3. Results

### 3.1. Clinical features

**Figure 1:**
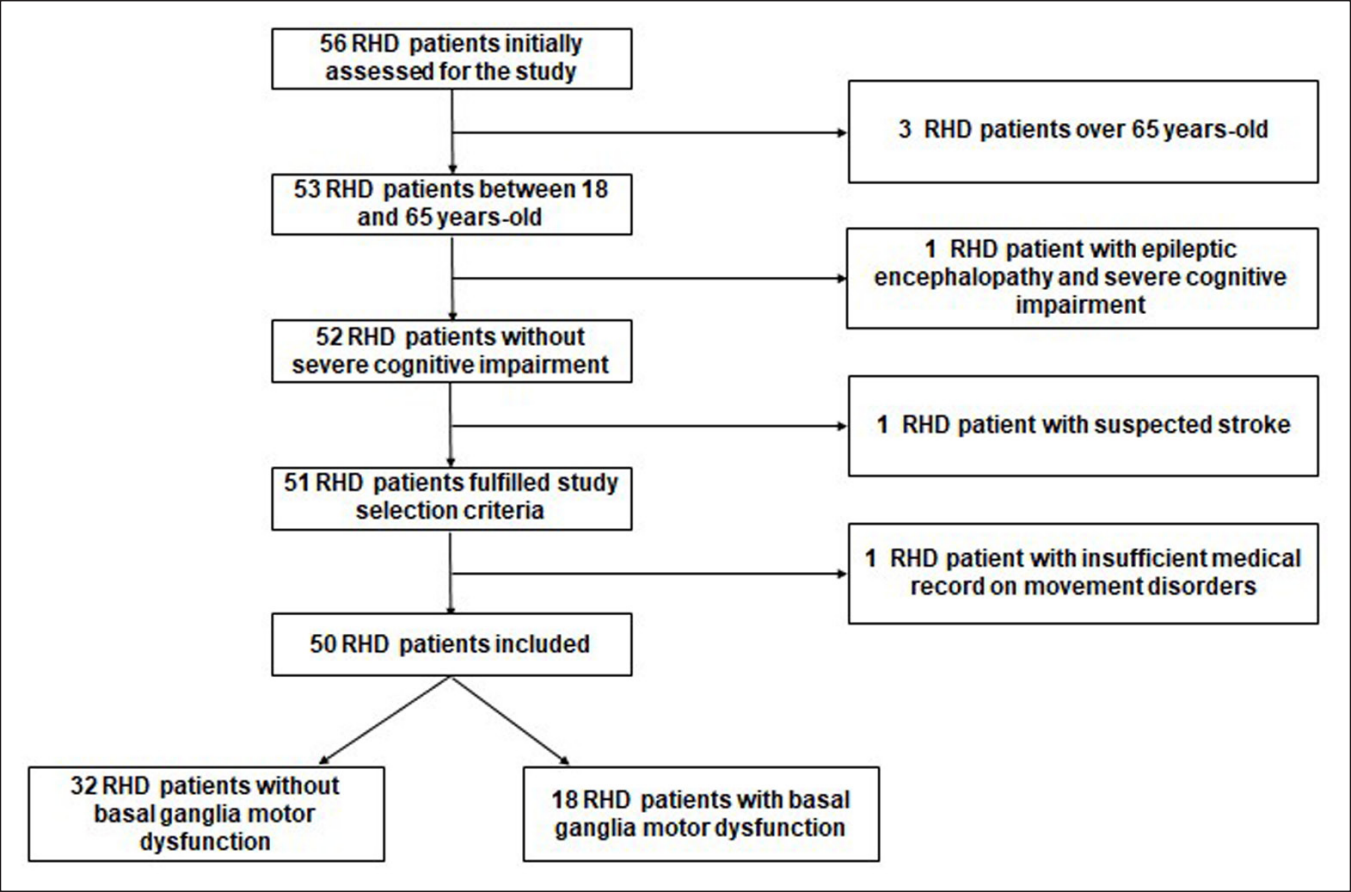
Flow chart of study population.

Demographic and clinical characteristics are summarized in Table 1. Among 56 patients with RHD initially invited to participate, 50 patients were enrolled in the study (Figure 1). Forty-two subjects (84%) were female, and the majority of patients were diagnosed with chronic RHD approximately nine years before the current assessment.

**Table 1:** Clinical, neurological and psychiatric features of the study population.


VARIABLES^*^		VALUE

Age (years)		43.2 ± 10.8

Female gender		42 (84)

Schooling (years)		8.5 ± 4.3

Time since diagnosis of rheumatic fever (years)		9.0 [5/22]

NYHA functional class	I/II	38 (76)

	III/IV	12 (24)

Systolic blood pressure (mmHg)		111 ± 14

Diastolic blood pressure (mmHg)		73 ± 10

**12-Item Short-Form Health Survey^†^**		

Physical Component Summary		45.5 ± 9.9

Mental Component Summary		44.4 ± 13.1

**Neurological and psychiatric characteristics**		

Current or history of chorea^‡^		24 (48)

Tics		9 (18)

Basal ganglia motor dysfunction^§^		18 (36)

Obsessive-compulsive symptoms		24 (48)

Major depressive disorder		17 (34)

Generalized anxiety disorder		27 (54)

Seizures		9 (18)

Migraine		26 (52)

Cognitive complaints^¶^		33 (66)


* Data are expressed as the mean value ± SD, median (interquartile range), or absolute numbers (percentage). ^†^ Revised form of the 12-Item Short-Form Health Survey. ^‡^ Patients with chorea observed in current neurologic examination plus patients with well-documented history of Sydenham’s chorea. ^§^ Basal ganglia motor dysfunction includes patients with well-documented diagnosis of Sydenham’s chorea and patients with current chorea and/or tics. ^¶^Patients with subjective complaints about difficulties in attention, memory and execution/planning of daily tasks.

At the time of assessment, severe functional limitation (NYHA functional class III–IV) was found in 24% of patients (Table 1). Mitral valve was affected in 47 patients (94%), with 46 patients (92%) previously been submitted to mitral valve intervention, mainly percutaneous mitral balloon commissurotomy (84%). Twenty-five patients (50%) presented with isolated mitral valve lesion, 21 (42%) had both mitral and aortic valves lesions, and one (2%) had the involvement of three valves, including tricuspid valve. Most patients (74%) had mixed mitral valve disease with predominance of mitral stenosis. Only three patients had aortic valve disease, predominantly aortic insufficiency.

Patients had low scores in both physical (45.5 ± 9.9) and mental components (44.4 ± 13.1) of SF-12v2, suggesting impairment of quality of life, but they were not associated with the severity of cardiac symptoms.

### 3.2. Neurological and psychiatric manifestations

Neurological and psychiatric symptoms were found in 41 (82%) patients. Nine patients (18%) did not present any neurological or psychiatric symptoms. Chorea was observed in 11 (22%) patients at examination and two other patients had a prior diagnosis of SC. Chronic tic disorders were present in 9 (18%) patients, and 4 (8%) of these patients also exhibited chorea. Therefore, clinically-determined BGMD was detected in 18 patients (36%) (Table 1). Seizures and subjective cognitive complaints were evident in 9 (18%) and 33 (66%) patients, respectively. Migraine was present in 26 (52%) patients. All patients who reported seizures had unrevealing brain CT or MRI scans.

OCS were observed in 24 (48%) patients, while major depressive disorder and generalized anxiety disorder was observed, respectively, in 17 (34%) and 27 (54%) patients. The main OCS were symmetry/ordering (22%), and contamination/cleaning (10%). Other OCS as damage or harmful thoughts (4%) and hoarding (2%) were less frequent. Five (10%) patients had two or more different OCS concomitantly. Psychosis was not found in the study population.

### 3.3. Variables associated with basal ganglia motor dysfunction

Demographic and clinical features according to BGMD are summarized in Table 2. Patients with BGMD were younger than patients without BGMD (p = 0.032), without differences regarding sex and years of schooling. No cardiologic or echocardiographic parameters were associated with BGMD (Table 2).

**Table 2:** Demographic and clinical features according to basal ganglia motor dysfunction.


CLINICAL PARAMETERS*	PATIENTS WITHOUT BASAL GANGLIA MOTOR DYSFUNCTION (N = 32)	PATIENTS WITH BASAL GANGLIA MOTOR DYSFUNCTION (N = 18)	P VALUE

Age (years)	45.7 ± 10.9	38.9 ± 9.4	0.032

Female gender (n/%)	26 (81)	16 (89)	0.694

Schooling (years)	8.5 ± 4.8	8.2 ± 3.5	0.685

NYHA class III–IV (n/%)	9 (28)	3 (17)	0.497

Valvar intervention (n/%)	30 (94)	16 (89)	0.612

Mitral valve area (cm^2^)	1.00 [0.83/1.40]	0.90 [0.77/1.14]	0.280

Left ventricular ejection fraction (%)	65 [57/69]	66 [62/69]	0.424

Left atrial volume (mL/m^2^)	55.5 [45/66]	51.3 [47.6/59.2]	0.534

Atrial fibrillation (n/%)	8 (25)	2 (11)	0.452

Mitral mean gradient (mmHg)	8.5 [6.5/14]	9.5 [8/13.7]	0.301

Moderate/severe MR (n/%)	2 (6)	1 (6)	1.000

Systolic pulmonary AP (mmHg)	37 [33/49]	44 [33/68]	0.275

**12-Item Short-Form Health Survey^§^**			

Physical Component Summary	44.3 ± 10.6	47.5 ± 8.5	0.275

Mental Component Summary	47.2 ± 13.5	39.5 ± 11.3	0.043


* Data are expressed as the mean value ± SD, median (interquartile range), or absolute numbers (percentage). ^§^ Revised form of the 12-Item Short-Form Health Survey. AP = artery pressure; MR = mitral regurgitation.

Neurological and psychiatric manifestations according to BGMD are shown in Table 3. The prevalence of OCS was much higher in patients with BGMD than those without (72% and 34%, respectively). Accordingly, higher scores on the Y-BOCS were associated with BGMD (p = 0.004). Major depressive disorder occurred in 56% of patients with BGMD whereas in only 22% of those without BGMD. Generalized anxiety disorder was also associated with BGMD (p = 0.018). There were no differences on the prevalence of prescribed antidepressants, anticonvulsants and benzodiazepines among patients with or without BGMD. No patient was using neuroleptics.

**Table 3:** Neurological and psychiatric disorders according to basal ganglia motor dysfunction.


NEUROLOGICAL AND PSYCHIATRIC CHARACTERISTICS*	PATIENTS WITHOUT BASAL GANGLIA MOTOR DYSFUNCTION (N = 32)	PATIENTS WITH BASAL GANGLIA MOTOR DYSFUNCTION (N = 18)	P VALUE

Obsessive-compulsive symptoms	11 (34)	13 (72)	0.018

Major depressive disorder	7 (22)	10 (56)	0.028

Generalized anxiety disorder	13 (41)	14 (78)	0.018

Seizures	4 (13)	5 (28)	0.253

Migraine	14 (44)	12 (67)	0.149

Cognitive complaints	22 (69)	11 (61)	0.757

**Prescribed Drugs**			

Antidepressants	3 (9)	2 (11)	1.000

Anticonvulsants	2 (6)	3 (19)	0.336

Benzodiazepines	0 (0)	2 (11)	0.125

**Validated Questionnaires**			

YBOCS score^†^	0 [0/10.8]	13 [0/24]	0.004

HADS ‡ Depression	6.4 ± 4.8	8.7 ± 5.1	0.115

Anxiety	7.6 ± 5.0	9.9 ± 4.6	0.102

MMSE Score^¶^	28 [25.5/30]	26 [24.8/29]	0.216

Low MMSE Score^£^	8 (25)	8 (44)	0.211

FAB score^§^	14.5 [12/16.8]	14 [13/15.3]	0.895


* Data are expressed as the mean value ± SD, median (interquartile range), or absolute numbers (percentage). ^†^ Yale-Brown obsessive-compulsive scale. ^‡^ Hospital Anxiety and Depression Scale. ^¶^ Mini-mental state examination. ^£^ MMSE scores below the cut-off proposed for Brazilian subjects, adjusted for schooling years. ^§^ Frontal assessment battery.

Migraine, cognitive complaints and seizures were frequent in the whole sample, not differing between groups. The score of the MCS of the SF-12v2 was significantly lower in patients with BGMD (p = 0.043), suggesting that neurological and behavioral symptoms influence the quality of life of these patients.

In a multivariate logistic regression analysis, age (adjusted odds ratio [OR]: 0.916, 95% confidence interval [CI]: 0.853 to 0.985; p = 0.018), major depressive disorder (adjusted OR: 7.534, 95% CI: 1.528 to 37.148; p = 0.013) and Y-BOCS (adjusted OR: 1.101, 95% CI: 1.022 to 1.187; p = 0.011) emerged as variables independently associated with BGMD (Table 4). Generalized anxiety disorder and migraine did not remain in the final model.

**Table 4:** Factors independently associated with basal ganglia motor dysfunction.


VARIABLES	BASAL GANGLIA MOTOR DYSFUNCTION	

	OR (95% CI)	P VALUE

Age	0.916 (0.853 – 0.985)	0.018

Major depressive disorder	7.534 (1.528 – 37.148)	0.013

Y–BOCS^†^	1.101 (1.022 – 1.187)	0.011


^†^ Yale-Brown obsessive-compulsive scale.

## 4. Discussion

The current study determined the prevalence of neurological and psychiatric disorders in patients with RHD, evidencing a high prevalence of persistent chorea and tics. While the severity of heart disease was not associated with quality of life and clinically-defined BGMD, the latter was associated with younger age, major depressive disorder, and higher Y-BOCS scores.

There is a significant concern about long-term complications of RHD since it represents an important cause of cardiovascular morbidity and mortality in young patients, especially in low-income countries [3]. However, there is limited information on persistent chorea and other neurological and behavioral manifestations in adult patients with RHD. The available studies focused on neurological and psychiatric disorders in acute RF or persistent symptoms in adolescents and young adults with SC [8,12,13,14,31]. This is the first study to investigate the prevalence of chorea and other neurological and behavioral disorders in middle-aged adults with RHD.

SC is present in 20 to 30% of patients with acute RF and is frequently reported as a benign and self-limited condition. However, around 50% of patients with SC have chorea lasting more than two years [32]. In the current study, all patients with RHD and chorea (n = 11, 22%) are presumably persistent SC since alternative causes of chorea were ruled out. The frequency of chorea of 22% in RHD is unexpectedly high since acute RF may have occurred as far as 20 to 30 years before. Interestingly, patients with BGMD were younger than those without BGMD, suggesting that the prevalence of chorea and tics may decrease over time. Future longitudinal studies must confirm this finding.

In acute RF, carditis may be present in up to 70% of patients with SC [5]. Although a well-recognized comorbidity, there are conflicting data on the potential association between SC and severity of carditis and development of RHD. While carditis was a marker of persistent chorea in a Brazilian study [32], Walker et al. (2007) reported that patients with carditis were less likely to display chorea, with a negative association between its severity and chorea [33]. A Turkish study could not establish any association between CS and RHD after following 12 SC patients with pure chorea for more than five years and not observing the development of RHD [34]. Our results did not show association between clinical and/or echocardiographic severity markers of RHD and BGMD, suggesting that motor signs, in other words chorea and tics, are not related to cardiac involvement. Since the severity of cardiac involvement is not necessarily associated with neurological signs indicating frontostriatal motor circuit impairment, the underlying pathophysiological processes of heart and brain dysfunction might be independent.

Behavioral disorders have been consistently associated with SC, notably OCS, depression, anxiety disorders [6]. There is also evidence that RF is associated with these disorders [5]. The landmark study of Swedo et al. (1989) described higher prevalence of OCS, anxiety and depressive symptoms among 23 SC patients compared to 14 RF patients without chorea [35]. Maia et al. (2005) confirmed a higher frequency of OCS in SC patients compared to those with RF and healthy controls [9]. In a more recent study, Moreira et al. (2014) reported that among 50 patients with SC consecutively evaluated, the most frequent psychiatric disorders were major depression (14%), generalized anxiety disorder (16%), social phobia (24%) and obsessive-compulsive disorder (OCD) (24%) [36]. They also reported a high prevalence of major depression in patients with persistent SC. Asbahr et al. (2004) found no difference in OCS prevalence between adult patients with RF and control patients with diabetes mellitus type 1 [12]. Conversely, Mercadante et al. (2000) reported that OCS were frequent in both RF patients with (22 patients) and without (20 patients) chorea [15]. In line with these findings, OCS have been reported to be more prevalent in 51 adult patients with RF and RHD (52.9%) compared to 46 control patients with non-rheumatic heart diseases (28.3%) [13]. We confirmed the high frequency of behavioral disorders in patients with RHD. The prevalence of OCS and major depressive disorder in our study were comparable to those reported in the literature for SC and RHD [9,13,36]. Generalized anxiety disorder was also highly prevalent, corroborating previous reports [15,36].

Cognitive functions may be impaired in SC patients, as observed in other primary basal ganglia disorders such as Huntington’s disease [8]. Individuals with SC may exhibit difficulties in executive functioning tasks and low performance in verbal fluency tests [6]. Adult patients who had chorea in childhood had worse performance in attention, speed information processing, executive functions and working memory tests compared to those who had RF without chorea [31]. Beato et al. (2010) reported that adult patients with both remitted and persistent SC had executive dysfunction, with no significant difference between both groups [8]. Decreased phonemic and semantic verbal fluency and impairment of verbal comprehension also occur in SC [10,11]. We also performed brief cognitive assessment with MMSE and FAB to investigate whether global cognition and executive function impairment were associated with BGMD. Patients with BGMD and those without BGMD had similar performance in FAB and MMSE. A similar performance in these instruments was also noticed in patients with and without subjective cognitive complaints.

Increased prevalence of migraine in school children with SC and RF was previously reported [7]. The frequency of migraine in children and adolescents with SC (21.8%) and RF (18.2%) was higher than the estimated prevalence for this age group (3.2 to 10.6%) [7]. Our study is the first to describe the prevalence of migraine in middle-aged adults with RHD/RF. The prevalence of migraine in our sample (52%) is much higher than the estimated prevalence of migraine in the general Brazilian population (around 15%) [37].

There are anecdotal reports of the association of seizures with SC. Ch’ien et al. (1978) described two patients with SC who presented focal impaired awareness seizures among 28 SC patients [38]. Moreover, 60% of the sample had unspecific electroencephalographic abnormalities. As another original contribution, we found a high prevalence of seizure history in RHD (18%). Since we excluded RHD patients with stroke, seizures cannot be seen as a complication of cerebral ischemia. As a word of caution, it is not possible to rule out that, in some patients, seizures could be triggered by unknown causes other than SC.

It is worth highlighting that low scores in MCS of the SF-12v2 were associated with BGMD, which suggests a potential negative impact of mental health on social and occupational functioning of patients with clinical signs of impairment of frontostriatal motor circuit.

### 4.1. Study limitations

The present study has several limitations. First, this study has a relatively small sample size, which might have underestimated the association of some variables, such as migraine, with BGMD. Conversely, we performed a comprehensive assessment of the patients, ruling out stroke and other conditions that might have inflated the results. Second, patients with RHD might represent a group of more severe RF, overestimating the prevalence of behavioral and neurological disorders. Nevertheless, RHD markers of severity did not associate with BGMD. Third, the high prevalence of female patients might have overestimated the prevalence of behavioral symptoms, but RHD is far more prevalent in women [1]. Fourth, seizures were very frequent in our population and we could not exclude other potential causes for epilepsy in all patients with seizures. Lastly, since this is a cross-sectional study, no cause-and-effect relationship can be inferred. A follow-up study is definitely warranted to elucidate the pattern of association/clustering and progression of these neurological and behavioral disorders over time and which factors are associated with their improvement and/or persistence.

## 5. Conclusion

Our study confirmed that neurological and behavioral disorders are remarkably frequent in middle-aged patients with RHD and may persist up to 20 to 30 years after RF, suggesting a persistent dysfunction of frontostriatal circuits. These disorders can also affect quality of life. The severity of heart disease was not related to the presence of clinical signs of BGMD, while younger age, major depressive disorder and OCS were. Similar to cardiac symptoms of RHD, such manifestations deserve a close attention from clinicians and researchers dealing with RF. Future studies are necessary for a better understanding of the factors and mechanisms underlying long-term behavioral and neurological symptoms persistence.
